# Birth Weight and Adult Obesity Index in Relation to the Risk of Hypertension: A Prospective Cohort Study in the UK Biobank

**DOI:** 10.3389/fcvm.2021.637437

**Published:** 2021-06-17

**Authors:** Yi Zhang, Jingjia Liang, Qian Liu, Xikang Fan, Cheng Xu, Aihua Gu, Wei Zhao, Dong Hang

**Affiliations:** ^1^State Key Laboratory of Reproductive Medicine, Institute of Toxicology, Nanjing Medical University, Nanjing, China; ^2^Department of Epidemiology and Biostatistics, Jiangsu Key Lab of Cancer Biomarkers, Prevention and Treatment, Collaborative Innovation Center for Cancer Personalized Medicine, School of Public Health, Nanjing Medical University, Nanjing, China; ^3^Jinling Hospital Department of Reproductive Medical Center affiliated School of Medicine, Nanjing University, Nanjing, China

**Keywords:** birth weight, adult obesity index, hypertension, blood pressure, epidemiology, prospective study

## Abstract

**Objectives:** To investigate the association between birth weight and the risk of hypertension, and to examine the interaction between birth weight and the adult obesity index.

**Methods:** We included 199,893 participants who had birth weight data and no history of hypertension at baseline (2006–2010) from the UK Biobank. A multivariate cubic regression spline was used to visually explore the dose-response relationship. Multivariate Cox proportional hazard regression models were used to calculate hazard ratios (HRs) and 95% confidence intervals (CIs).

**Results:** We observed a nonlinear inverse association between birth weight and hypertension. The risk for hypertension decreased as birth weight increased up to approximately 3.80 kg. Compared with the participants with the fourth quintile of birth weight (3.43–3.80 kg), those with the first quartile of birth weight (<2.88 kg) were associated with a 25% higher risk of hypertension [HR 1.25; 95% CI (1.18–1.32)]. In addition, the participants with birth weight <2.88 kg and body mass index ≥30 kg/m^2^ had the highest risk [HR 3.54; 95% CI (3.16–3.97); *p* for interaction <0.0001], as compared with those with birth weight between 3.43–3.80 kg and body mass index between 18.5–25.0 kg/m^2^. These associations were largely consistent in the stratified and sensitivity analyses.

**Conclusion:** Our findings indicate that lower birth weight is nonlinearly correlated with higher risk of hypertension, and birth weight between 3.43–3.80 kg might represent an intervention threshold. Moreover, lower birth weight may interact with adult obesity to significantly increase hypertension risk.

## Introduction

Fetal malnutrition, the primary indicator of which is low birth weight, can permanently alter organ structure and function in a way that predisposes the offspring to cardiovascular disease (CVD) in adulthood (1, 2). Growing evidence has suggested that low birth weight increases the risk of hypertension (3–5).

Although some epidemiological studies have suggested a nonlinear inverse association between birth weight and hypertension (6–9), the intervention threshold of birth weight for hypertension remains undetermined. A previous meta-analysis including 4,335,149 participants suggested that those with birth weight of 4.0–4.5 kg had the lowest risk (10). However, the meta-analysis contained significant heterogeneity that limited the validity of summary estimates, and the ability to control for potential confounders (e.g., maternal smoking) was insufficient. In addition, previous results are mixed regarding the association between high birth weight and hypertension risk. Some studies reported higher birth weight in relation to increased hypertension risk (11–13), whereas other studies did not find such an association (14, 15). These conflicting results might be attributed to the differences in sample sizes and confounder adjustments (13). Furthermore, low birth weight and obesity in adulthood can both stimulate the sympathetic nervous system and alter renal function (16, 17). Few studies have examined potential interaction between birth weight and the adult obesity index on hypertension risk, although there is evidence showing an effect on CVD risk (18, 19).

Therefore, for the current study we used data from the UK Biobank, a large prospective cohort study conducted in UK, to investigate the dose-response association of birth weight with hypertension and blood pressure. Moreover, we examined potential interactions between birth weight and the adult obesity index.

## Methods and Materials

### Study Population

The UK Biobank is a prospective cohort study recruiting half a million participants aged 37–73 years between 2006 and 2010 (20). At baseline, participants were asked to provide electronically signed consent, answer touch-screen questionnaires, and complete physical and anthropometric measurements. Follow-up with the UK Biobank was performed on an ongoing basis through study visits and linkage to national health records, death registers, and primary care records, as described previously (21). The UK Biobank has received ethical approvals from the UK Biobank Research Ethics Committee and Human Tissue Authority.

In the current study, we excluded participants who self-reported a history of hypertension or were taking antihypertensive medication at baseline (*n* = 147,989). We further excluded 151,078 individuals with missing birth weight data and 3,661 individuals with missing values on the main covariables. Finally, a total of 199,893 participants were included (Supplementary Figure 1). The basic characteristics of the excluded participants were similar to those included (Supplementary Table 1).

### Birth Weight and Physical Measurements

Participants were asked to report their own birth weight (either in kilograms directly, or in imperial pounds and ounces). Standing height was measured using a Seca202 device. Body mass index (BMI) was defined as weight divided by height squared (m^2^). Waist and hip circumferences were measured with a Seca200 measuring tape using standard procedures, and waist-to-hip ratio (WHR) was the ratio of waist circumference to hip circumference.

Systolic blood pressure (SBP) and diastolic blood pressure (DBP) measurements were taken in a seated position after a few minutes of rest using an Omron 705 IT electronic blood pressure monitor. A manual sphygmometer was used if the standard automated device could not be employed. Further details of these measurements can be found in the UK Biobank online protocol (https://biobank.ndph.ox.ac.uk/showcase/showcase/docs/Bloodpressure.pdf" https://biobank.ndph.ox.ac.uk/showcase/showcase/docs/Bloodpressure.pdf). Means of SBP and DBP from two automated or two manual blood pressure measurements were calculated.

### Assessment of Hypertension

The date and cause of hospital admissions were confirmed by electronic health records linkage to health episode statistics (England and Wales) and Scottish morbidity records (Scotland). Date of death was obtained from death certificates held by the National Health Service Information Center (England and Wales) and the National Health Service Central Register Scotland (Scotland) (22). The primary outcome was incident hypertension, which was defined according to the International Classification of Diseases edition 10 (ICD-10) codes I10-I15.

### Statistical Analysis

Person-time of follow-up was calculated for each participant from the age in months at the return date of the baseline questionnaire (2006–2010) until the age in months at the date of first diagnosis of hypertension or the end of follow-up (November 30, 2016, in Edinburgh, Scotland, and January 31, 2018, in England or Wales), whichever came first. Cox proportional hazards regression models with age as the time scale were used to analyze the association between birth weight and hypertension, reporting hazard ratio (HR) and 95% confidence intervals (CI).

A multivariate restricted cubic spline with four knots was used to visually explore the nonlinear association of birth weight with hypertension. We used a likelihood ratio test (LRT) to compare the model with only the linear term of birth weight to the model with both the linear and the cubic spline terms, with a *p*-value < 0.05 denoting significant nonlinearity.

In the multivariate analysis of hypertension, we adjusted for age, sex, and early life events, including maternal smoking, breastfeeding, part of a multiple birth, and birthplace (Model 1). We also further adjusted for the Townsend deprivation index, college or university degree, BMI, summed metabolic equivalent of task hours per week (MET-hours/week) for physical activity, smoking status, alcohol intake frequency, intake of vegetables and fruit, family history of hypertension, and prevalence of diabetes (Model 2). Multivariate linear regression analysis was conducted to examine the associations between birth weight and SBP and DBP separately (*n* = 199,765). Birth weight was treated as a categorical (the quintiles) or continuous (per SD increment) variable.

Stratified analyses were conducted according to sex (male, female), maternal smoking (yes, no), breastfeeding (yes, no), physical activity (<median, ≥median), and smoking status (never, former, current). We tested the interaction between birth weight and each of the stratification variables using LRT, comparing a model with and without interaction terms.

In the joint analysis of birth weight and BMI, the fourth quintile of birth weight (3.43–3.80 kg) and BMI in adulthood between 18.5–25.0 kg/m^2^ was treated as the reference group. *P* for interaction was assessed by a Wald test for the cross-product terms between birth weight and BMI (continuous). Sensitivity analyses were performed by excluding individuals who were part of multiple births (*n* = 5,431).

All statistical tests were two-sided, and SAS version 9.4 (SAS Institute) was used for all analyses. *P* < 0.05 was defined as statistically significant.

## Results

During a median follow-up of 8.8 (interquartile range: 8.1–9.4) years, 12,333 hypertension events occurred among 199,893 participants. The basic characteristics of participants according to the quintile of birth weight are shown in Table 1. Those with lower birth weight were more likely to be part of a multiple birth and exposed to maternal smoking around birth, and were less likely to be breastfed.

**Table 1:** Basic characteristics of study participants according to birth weight.

**Characteristics**	**Birth weight quintiles**				
	**Q1 (<2.88kg)**	**Q2 (2.88–3.18kg)**	**Q3 (3.19–3.42kg)**	**Q4 (3.43-3.80 kg)**	**Q5 (>3.80 kg)**
Participants, No. (%)	39,042 (19.53)	43,780 (21.90)	36,948 (18.48)	40,493 (20.26)	39,630 (19.83)
**Perinatal**					
Female, No. (%)	28,740 (73.61)	29,709 (67.86)	23,790 (64.39)	23,623 (58.34)	20,757 (52.38)
White, No. (%)	37,252 (95.70)	42,165 (96.56)	36,104 (97.92)	39,399 (97.52)	38,674 (97.80)
Part of a multiple birth, No. (%)	3,880 (9.95)	806 (1.84)	366 (0.99)	228 (0.56)	151 (0.38)
Breastfeeding, No. (%)	20,987 (60.98)	27,614 (69.76)	24,015 (70.23)	26,538 (71.44)	25,581 (72.49)
Maternal smoking around birth, No. (%)	11,988 (34.32)	11,577 (29.33)	9,167 (27.43)	9,260 (25.26)	8,843 (24.83)
**Baseline**					
Age, mean (SD), year	54.32 (8.09)	54.32 (8.13)	53.71 (7.87)	53.30 (7.96)	54.41 (8.13)
Townsend deprivation index <0, No. (%)	28,312 (72.52)	32,683 (74.65)	27,611 (74.73)	30,302 (74.83)	29,517 (74.48)
BMI, mean (SD), kg/m^2^	26.39 (4.57)	26.25 (4.35)	26.39 (4.34)	26.60 (4.40)	27.06 (4.49)
Physical activity, mean (SD), MET-hours/week	44.85 (44.71)	44.62 (44.09)	44.57 (44.23)	44.26 (44.52)	45.79 (46.00)
Current smoking, No. (%)	3,985 (10.24)	4,246 (9.72)	3,875 (10.52)	4,116 (10.19)	4,325 (10.95)
Alcohol intake daily or almost daily, No. (%)	6,291 (16.12)	8,360 (19.11)	7,313 (19.81)	8,157 (20.16)	8,421 (21.26)
Family history of hypertension, No. (%)	19,139 (52.42)	21,099 (51.13)	18,251 (52.08)	19,469 (50.89)	18,341 (49.40)
Vegetable intake, mean (SD), serving/day	2.25 (2.13)	2.23 (2.14)	2.24 (2.09)	2.23 (2.08)	2.23 (2.21)
Fruit intake, mean (SD), serving/day	2.24 (1.57)	2.23 (1.51)	2.24 (1.53)	2.23 (1.54)	2.25 (1.57)
DBP, mean (SD), mmHg	80.89 (9.74)	80.52 (9.68)	80.57 (9.60)	80.50 (9.60)	80.73 (9.57)
SBP, mean (SD), mmHg	134.32 (17.94)	133.48 (17.60)	132.89 (17.06)	132.42 (17.01)	133.24 (16.93)

*BMI, body mass index (calculated as weight in kilograms divided by height in meters squared); MET, metabolic equivalent of task; DBP, diastolic blood pressure; SBP, systolic blood pressure; SD, standard deviation*.

**Figure 1:**
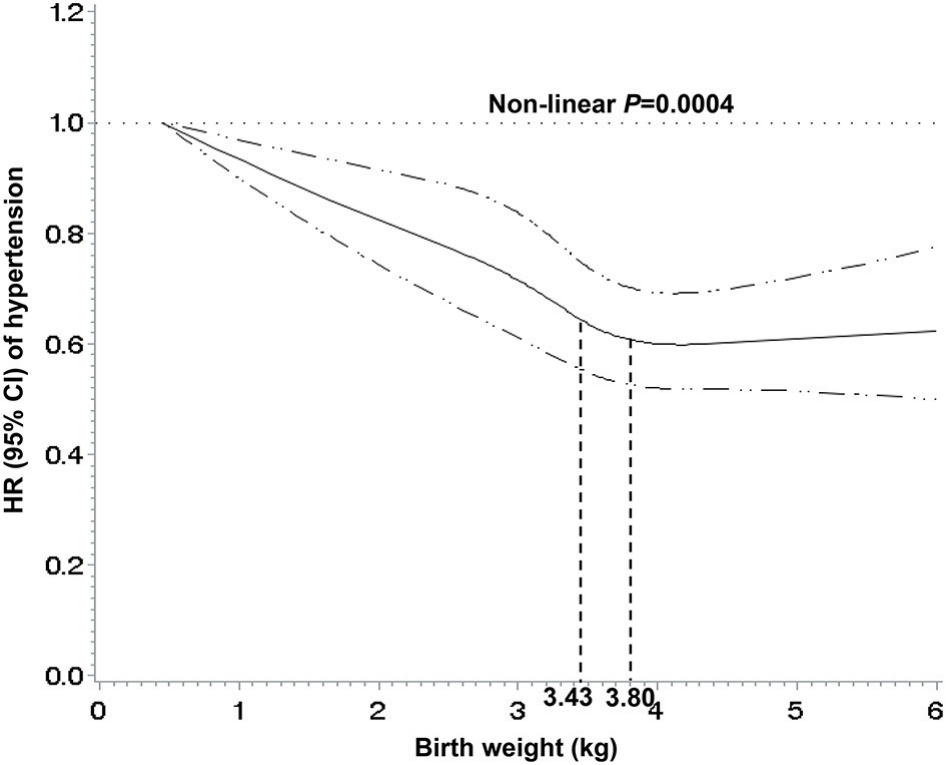
Nonlinear associations between birth weight and risk of hypertension. The associations were examined by multivariate Cox regression models based on restricted cubic splines. Participants with birth weight >6 kg were not included (*n* = 147). Solid line represents estimates of hazard ratios and dashed line represents 95% confidence intervals.

A nonlinear inverse association between birth weight and hypertension is observed in Figure 1. The risk of hypertension decreased as birth weight increased up to approximately 3.80 kg (*p* for nonlinearity = 0.0004).

In the fully adjusted models, compared with the fourth quintile of birth weight (3.43–3.80 kg), the first quartile (<2.88 kg) was associated with a 25% higher risk of hypertension [HR 1.25; 95% CI (1.18–1.32)] (Table 2).

**Table 2:** Associations between birth weight and hypertension.

**Hypertension**	**Birth weight quintiles, HR (95% CI)**					***P* for non-linearity**
	**Q1 (<2.88 kg)**	**Q2 (2.88–3.18 kg)**	**Q3 (3.19–3.42 kg)**	**Q4 (3.43–3.80 kg)**	**Q5 (>3.80 kg)**	
No. of cases/ person years	2,803/332,781.46	2,729/374,124.06	2,115/317,483.58	2,247/347,241.82	2,439/339,481.78	NA
Model 1^a^	1.27 (1.20–1.35)	1.08 (1.02–1.15)	1.03 (0.97–1.09)	Ref (1)	1.00 (0.94–1.06)	<0.0001
Model 2^b^	1.25 (1.18–1.32)	1.11 (1.05–1.17)	1.04 (0.98–1.11)	Ref (1)	0.95 (0.9–1.01)	0.0004

*HR, hazard ratio; CI, confidence interval; Ref, reference; NA, not applicable*.

a *Model 1: adjusted for age, sex, maternal smoking, breastfeeding, birth place, part of a multiple birth*.

b *Model 2: model 1 plus Townsend deprivation index, college or university degree, body mass index, physical activity (MET-hours/week), smoking status, alcohol intake frequency, intake of vegetables and fruit, family history of hypertension, prevalent diabetes*.

Table 3 shows the association between birth weight and blood pressure. A unit increase per SD increment in birth weight was associated with lower SBP [β coefficient = −1.07; 95% CI (−1.14 to −1.00)] and lower DBP [β coefficient = −0.47; 95% CI (−0.51 to −0.44)].

**Table 3:** Associations between birth weight and blood pressure.

**Blood pressure**	**Birth weight quintiles**, ***β*** **coefficient (95% CI)**					**Per SD increase β coefficient (95% CI)**
	**Q1 (<2.88 kg)**	**Q2 (2.88–3.18 kg)**	**Q3 (3.19–3.42 kg)**	**Q4 (3.43–3.80 kg)**	**Q5 (>3.80 kg)**	
No. (%)	39,005 (19.53)	43,754 (21.90)	36,923 (18.48)	40,468 (20.26)	39,615 (19.83)	NA
**SBP**						
Model 1^a^	2.27 (2.04 to 2.49)	1.03 (0.81 to 1.24)	0.62 (0.39 to 0.85)	Ref (0)	−0.4 (−0.62 to −0.18)	−0.93 (−1.01 to −0.86)
Model 2^b^	2.36 (2.14 to 2.58)	1.23 (1.01 to 1.44)	0.72 (0.5 to 0.94)	Ref (0)	−0.69 (−0.9 to −0.47)	−1.07 (−1.14 to −1.00)
**DBP**						
Model 1^a^	0.91 (0.78 to 1.04)	0.31 (0.18 to 0.44)	0.28 (0.14 to 0.41)	Ref (0)	−0.11 (−0.24 to 0.02)	−0.36 (−0.40 to −0.32)
Model 2^b^	0.99 (0.86 to 1.11)	0.50 (0.37 to 0.62)	0.38 (0.25 to 0.50)	Ref (0)	−0.36 (−0.49 to −0.24)	−0.47 (−0.51 to −0.44)

*CI, confidence interval; Ref, reference; NA, not applicable; DBP, diastolic blood pressure; SBP, systolic blood pressure*.

a *Model 1: adjusted for age, sex, maternal smoking, breastfeeding, birth place, part of a multiple birth*.

b *Model 2: model 1 plus Townsend deprivation index, college or university degree, body mass index, physical activity (MET-hours/week), smoking status, alcohol intake frequency, intake of vegetables and fruit, family history of hypertension, prevalent diabetes. SD = 0.65*.

In the stratified analysis (Supplementary Figure 2), the association between birth weight and hypertension was largely consistent across subgroups, except that the association was stronger in females (*p* for interaction = 0.004).

**Figure 2:**
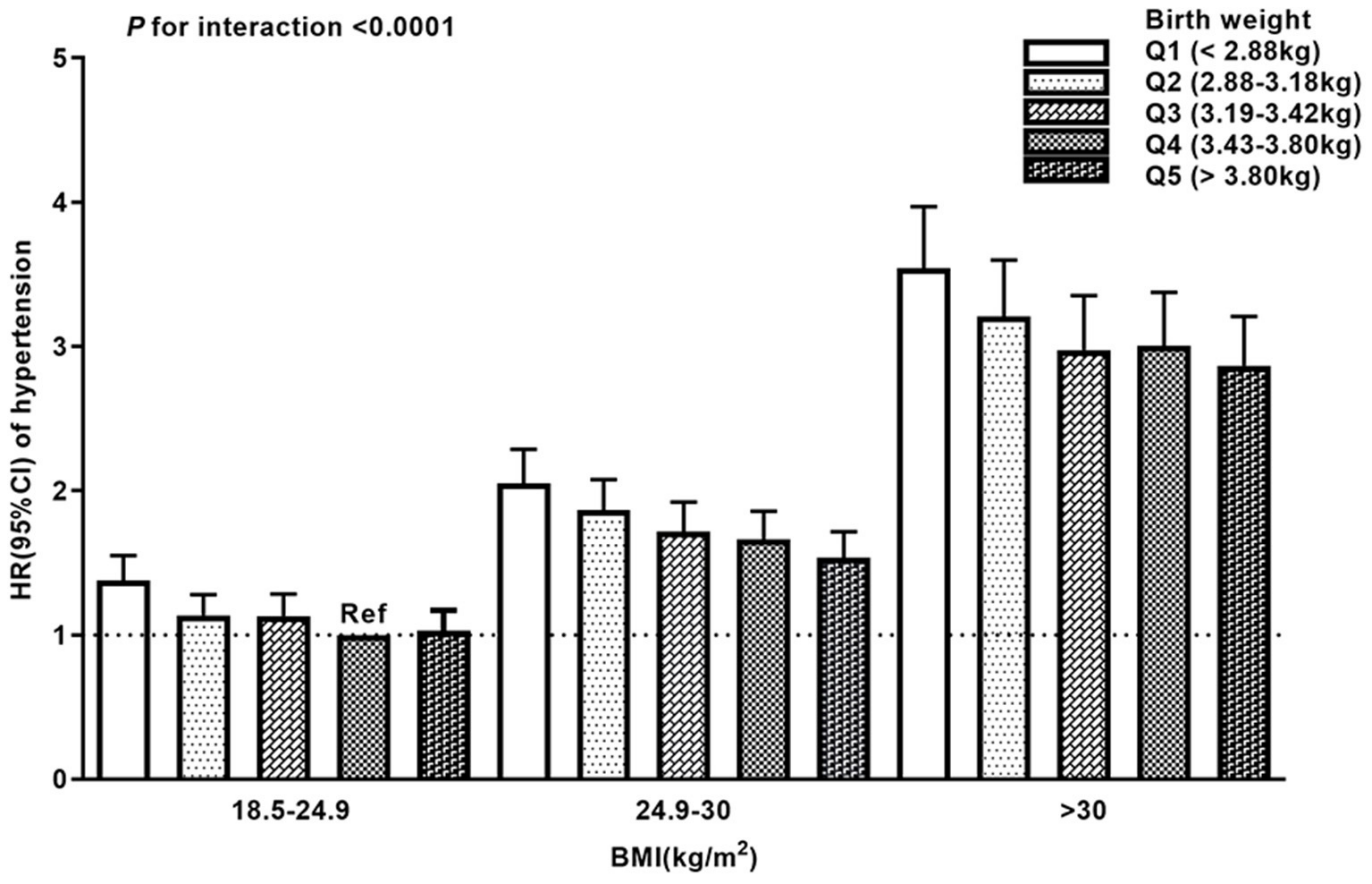
Joint analysis of birth weight and body mass index (BMI) in the full model. The participants with birth weight in group Q4 (3.43–3.80 kg) and BMI in adult between 18.5–25.0 kg/m^2^ were treated as the reference group (Ref). Error bars represent 95% confidence intervals of the hazard ratios.

A multiplicative interaction between birth weight and obesity index was observed in the joint analysis for birth weight and adult BMI in relation to hypertension risk (Figure 2). Compared with the reference group (birth weight between 3.43–3.80 kg and BMI between 18.5–25 kg/m^2^), participants with low birth weight and adult obesity (birth weight <2.88 kg and BMI ≥30 kg/m^2^) had the highest risk of hypertension [HR 3.54; 95% CI (3.16, 3.97); *p* for interaction <0.0001]. A similar pattern of correlations was observed between birth weight, WHR, and hypertension (Supplementary Figure 3).

Sensitivity analysis by excluding 5,431 participants who were part of a multiple birth showed similar associations between birth weight and hypertension (Supplementary Table 2).

## Discussion

In this large prospective cohort study, we observed a nonlinear association between birth weight and hypertension risk. Moreover, we observed a multiplicative interaction between birth weight and obesity index, and participants with low birth weight and adult obesity had the highest risk of hypertension.

Most previous studies investigating the association between birth weight and hypertension risk were based on predefined categories of birth weight. The lowest risk for hypertension was observed in different ranges of birth weight, such as 2.5–3.5 kg, ≥4.5 kg, 4.0–4.5 kg, and ≥4.0 kg (5, 6, 10, 23). A multivariate restricted cubic spline can display more information than the categorical data, and in the current study a nonlinear inverse association was found between birth weight and hypertension. The risk of hypertension decreased as birth weight increased, up to approximately 3.80 kg. Our data indicated that higher birth weight did not increase the hypertension risk, in line with the results from previous studies (14, 24). The World Health Organization defined low birth weight as <2.50 kg (25). The cutoff was based on epidemiological observations that infants with birth weight <2.50 kg had a higher mortality risk than ≥2.50 kg (26). Nevertheless, it may not be the optimal target for mitigating the increasing epidemic of hypertension. Our findings suggested that birth weight between 3.43–3.80 kg might represent a potential threshold for reducing hypertension risk.

Epidemiological evidence has shown an association between low birth weight and obesity in adulthood (27). Consistently, we found that individuals with lower birth weight were more likely to have a higher BMI or waist-to-hip ratio in the UK biobank (data not shown). When we adjust for potential confounders, including BMI or waist-to-hip ratio, the association between birth weight and hypertension remained stable. We also found an inverse association between birth weight and blood pressure, in line with the results from previous observational studies (3, 28, 29). Furthermore, a Mendelian randomization study, which took advantage of genetic variants as instrumental variables for birth weight, supported an inverse association between birth weight and blood pressure (30). However, several studies reported that birth weight was inversely associated with SBP only (31, 32). This might be attributed to the limited statistical power of these studies to identify the relatively small effects of birth weight on DBP (31). The current study confirmed that the magnitude of the association for DBP was smaller than that for SBP.

Joint analysis indicated that participants with low birth weight and adult obesity had the highest risk of hypertension. Consistently, previous studies have shown that low-birth-weight children with obesity now tend to have higher values of systolic blood pressure than those who are obese with normal birth weight (33, 34). It is plausible that individuals with an abnormal intrauterine environment (such as poor nutrition) are more sensitive to the adverse effects of adult obesity on hypertension risk. Our study is the first to suggest that low birth weight might interact with adult obesity to increase hypertension risk in adult life. Targeting interventions and prevention of obesity, especially for those with low birth weight, might be given high priority.

Biological mechanisms by which low birth weight affects the development of hypertension are complex and remain equivocal. The proposed mechanisms are mostly related to reduced nephron counts, sympathetic hyperactivity, and impairment of vascular structure and function (17). In addition, intrauterine growth restriction may lead to impaired function of the hypothalamic-pituitary-adrenal axis and increased activity of renin angiotensin aldosterone system, thereby elevating the risk for hypertension (35, 36). Animal studies have also found that fetal exposure to maternal protein restriction lead to an increase in renal inflammation, indicating an important role of inflammatory processes caused by low birth weight in the development of hypertension (37).

## Strengths and Limitations of the Study

Our study has several strengths, including a large sample size, long-term follow-up, and strict adjustment for potential confounders. We also estimated the joint effects of birth weight and adult obesity. Several limitations need to be acknowledged (12). First, early life exposures were based on self-reporting, which could lead to recall bias. However, we calculated the weighted Kappa correlation coefficients of self-reported birth weight between baseline and first (*n* = 12,171) and second follow-up (*n* = 4,272), which were 0.82 and 0.81, respectively, indicating a good reliability of self-reported birth weight in the UK biobank. In addition, a previous study of the UK Biobank estimated the associations of self-reported birth weight with sex, deprivation index, multiple births, and maternal smoking, and supported the validity of self-reported birth weight (38). Some other studies also confirmed the validity of self-reported birth weight by comparing it with mothers' recall or hospital birth records (39, 40). Second, residual confounding could still exist in the observational study, and we were thus unable to make causal inferences. Finally, most of the UK Biobank participants were of caucasian race, which limits the generalizability of our findings to other ethnic populations.

## Conclusions

The current study indicates a nonlinear inverse association between birth weight and hypertension and suggests birth weight between 3.43–3.80 kg as a threshold for reducing disease risk. Low birth weight may interact with adult obesity to increase hypertension risk in adult life. Further studies are warranted to explore underlying mechanisms.

## Data Availability Statement

The raw data supporting the conclusions of this article will be made available by the authors, without undue reservation.

## Author Contributions

DH, AG, and WZ contributed to the conception and design of the study. YZ and DH have full access to all the data in the study and take responsibility for the integrity of the data and the accuracy of the data analysis. YZ, JL, and QL did the statistical analysis and drafted the manuscript. XF and CX critically revised the manuscript for important intellectual content. All authors reviewed and approved the final manuscript.

## Conflict of Interest

The authors declare that the research was conducted in the absence of any commercial or financial relationships that could be construed as a potential conflict of interest.

## Abbreviations

BMI: body mass index
CVD: cardiovascular disease
WHR: waist-to-hip ratio
SBP: systolic blood pressure
DBP: diastolic blood pressure
LRT: likelihood ratio test
MET: metabolic equivalent of task
SD: standard deviation
HR: hazard ratio
CI: confidence interval
Ref: reference
NA: not applicable.

## References

[1] Miranda JO Ramalho C Henriques-Coelho T Areias JC. Fetal programming as a predictor of adult health or disease: the need to reevaluate fetal heart function. Heart Fail Rev. (2017) 22:861–77. 10.1007/s10741-017-9638-z 28730459

[2] Lane RH. Fetal programming, epigenetics, and adult onset disease. Clin Perinatol. (2014) 41:815–31. 10.1016/j.clp.2014.08.006 25459776

[3] Chen W Srinivasan SR Berenson GS. Amplification of the association between birthweight and blood pressure with age: the Bogalusa Heart Study. J Hypertens. (2010) 28:2046–52. 10.1097/HJH.0b013e32833cd31f 20616754 PMC3105358

[4] Huxley R Neil A Collins R. Unravelling the fetal origins hypothesis: is there really an inverse association between birthweight and subsequent blood pressure? Lancet. (2002) 360:659–65. 10.1016/S0140-6736(02)09834-3 12241871

[5] Li Y Ley SH VanderWeele TJ Curhan GC Rich-Edwards JW Willett WC. Joint association between birth weight at term and later life adherence to a healthy lifestyle with risk of hypertension: a prospective cohort study. BMC Med. (2015) 13:175. 10.1186/s12916-015-0409-1 26228391 PMC4521367

[6] Xia Q Cai H Xiang YB Zhou P Li H Yang G. Prospective cohort studies of birth weight and risk of obesity, diabetes, and hypertension in adulthood among the Chinese population. J Diabetes. (2019) 11:55–64. 10.1111/1753-0407.12800 29893042 PMC6334524

[7] Andersson SW Lapidus L Niklasson A Hallberg L Bengtsson C Hulthen L. Blood pressure and hypertension in middle-aged women in relation to weight and length at birth: a follow-up study. J Hypertens. (2000) 18:1753–61. 10.1097/00004872-200018120-00008 11132598

[8] Pocobelli G Dublin S Enquobahrie DA Mueller BA. Birth weight and birth weight for gestational age in relation to risk of hospitalization with primary hypertension in children and young adults. Matern Child Health J. (2016) 20:1415–23. 10.1007/s10995-016-1939-7 26979614 PMC5491096

[9] Curhan GC Chertow GM Willett WC Spiegelman D Colditz GA Manson JE. Birth weight and adult hypertension and obesity in women. Circulation. (1996) 94:1310–5. 10.1161/01.CIR.94.6.1310 8822985

[10] Knop MR Geng TT Gorny AW Ding R Li C Ley SH. Birth weight and risk of type 2 diabetes mellitus, cardiovascular disease, and hypertension in adults: a meta-analysis of 7 646 267 participants from 135 studies. J Am Heart Assoc. (2018) 7:e008870. 10.1161/JAHA.118.008870 30486715 PMC6405546

[11] Tian JY Cheng Q Song XM Li G Jiang GX Gu YY. Birth weight and risk of type 2 diabetes, abdominal obesity and hypertension among Chinese adults. Eur J Endocrinol. (2006) 155:601–7. 10.1530/eje.1.02265 16990660

[12] Bowers K Liu G Wang P Ye T Tian Z Liu E. Birth weight, postnatal weight change, and risk for high blood pressure among Chinese children. Pediatrics. (2011) 127:e1272–9. 10.1542/peds.2010-2213 21502227 PMC3387869

[13] Kuciene R Dulskiene V Medzioniene J. Associations between high birth weight, being large for gestational age, and high blood pressure among adolescents: a cross-sectional study. Eur J Nutr. (2018) 57:373–81. 10.1007/s00394-016-1372-0 28058464 PMC5847040

[14] Johnsson IW Haglund B Ahlsson F Gustafsson J. A high birth weight is associated with increased risk of type 2 diabetes and obesity. Pediatr Obes. (2015) 10:77–83. 10.1111/ijpo.230 24916852

[15] Wei JN Li HY Sung FC Lin CC Chiang CC Li CY. Birth weight correlates differently with cardiovascular risk factors in youth. Obesity. (2007) 15:1609–16. 10.1038/oby.2007.190 17557999

[16] Seravalle G Grassi G. Obesity and hypertension. Pharmacol Res. (2017) 122:1–7. 10.1016/j.phrs.2017.05.013 28532816

[17] Alexander BT Dasinger JH Intapad S. Fetal programming and cardiovascular pathology. Comprehen Physiol. (2015) 5:997–1025. 10.1002/cphy.c140036 PMC4772789 25880521

[18] Tian J Qiu M Li Y Zhang X Wang H Sun S. Contribution of birth weight and adult waist circumference to cardiovascular disease risk in a longitudinal study. Sci Rep. (2017) 7:9768. 10.1038/s41598-017-10176-6 28852140 PMC5575020

[19] Lawlor DA Ronalds G Clark H Smith GD Leon DA. Birth weight is inversely associated with incident coronary heart disease and stroke among individuals born in the 1950s: findings from the Aberdeen Children of the 1950s prospective cohort study. Circulation. (2005) 112:1414–8. 10.1161/CIRCULATIONAHA.104.528356 16129799

[20] Collins R. What makes UK Biobank special? Lancet. (2012) 379:1173–4. 10.1016/S0140-6736(12)60404-8 22463865

[21] Honigberg MC Patel AP Lahm T Wood MJ Ho JE Kohli P. Association of premature menopause with incident pulmonary hypertension: a cohort study. PLoS ONE. (2021) 16:e0247398. 10.1371/journal.pone.0247398 33690615 PMC7946190

[22] Sudlow C Gallacher J Allen N Beral V Burton P Danesh J. UK biobank: an open access resource for identifying the causes of a wide range of complex diseases of middle and old age. PLoS Med. (2015) 12:e1001779. 10.1371/journal.pmed.1001779 25826379 PMC4380465

[23] Tan M Cai L Ma J Jing J Ma Y Chen Y. The association of gestational age and birth weight with blood pressure among children: a Chinese national study. J Hum Hypertens. (2018) 32:651–9. 10.1038/s41371-018-0084-8 29942098

[24] Dong YH Zou ZY Yang ZP Wang ZH Jing J Luo JY. Association between high birth weight and hypertension in children and adolescents: a cross-sectional study in China. J Hum Hypertens. (2017) 31:737–43. 10.1038/jhh.2017.22 28382956

[25] Blencowe H Krasevec J de Onis M Black RE An X Stevens GA. National, regional, and worldwide estimates of low birthweight in 2015, with trends from 2000: a systematic analysis. Lancet Global Health. (2019) 7:e849–e60. 10.1016/S2214-109X(18)30565-5 31103470 PMC6560046

[26] Kramer MS. Determinants of low birth weight: methodological assessment and meta-analysis. Bull World Health Org. (1987) 65:663–737. 3322602 PMC2491072

[27] Barker DJ. The developmental origins of chronic adult disease. Acta Paediatr Suppl. (2004) 93:26–33. 10.1111/j.1651-2227.2004.tb00236.x 15702667

[28] Bustos P Amigo H Bangdiwala SI Pizarro T Rona RJ. Does the association between birth weight and blood pressure increase with age? A longitudinal study in young adults. J Hypertens. (2016) 34:1062–7. 10.1097/HJH.0000000000000912 27077730

[29] Hovi P Vohr B Ment LR Doyle LW McGarvey L Morrison KM. Blood pressure in young adults born at very low birth weight: adults born preterm international collaboration. Hypertension. (2016) 68:880–7. 10.1161/HYPERTENSIONAHA.116.08167 27572149

[30] Warrington NM Beaumont RN Horikoshi M Day FR Helgeland Ø Laurin C. Maternal and fetal genetic effects on birth weight and their relevance to cardio-metabolic risk factors. Nat Genet. (2019) 51:804–14. 10.1038/s41588-019-0403-1 31043758 PMC6522365

[31] Hardy R Wadsworth ME Langenberg C Kuh D. Birthweight, childhood growth, and blood pressure at 43 years in a British birth cohort. Int J Epidemiol. (2004) 33:121–9. 10.1093/ije/dyh027 15075157

[32] Järvelin MR Sovio U King V Lauren L Xu B McCarthy MI. Early life factors and blood pressure at age 31 years in the 1966 northern Finland birth cohort. Hypertension. (2004) 44:838–46. 10.1161/01.HYP.0000148304.33869.ee 15520301

[33] Lurbe E Carvajal E Torro I Aguilar F Alvarez J Redon J. Influence of concurrent obesity and low birth weight on blood pressure phenotype in youth. Hypertension. (2009) 53:912–7. 10.1161/HYPERTENSIONAHA.109.129155 19414646

[34] Leon DA Koupilova I Lithell HO Berglund L Mohsen R Vagero D. Failure to realise growth potential in utero and adult obesity in relation to blood pressure in 50 year old Swedish men. BMJ. (1996) 312:401–6. 10.1136/bmj.312.7028.401 8601110 PMC2350092

[35] Rasyid H Bakri S. Intra-uterine growth retardation and development of hypertension. Acta Med Indones. (2016) 48:320–4. https://pubmed.ncbi.nlm.nih.gov/28143994/28143994

[36] Seckl JR. Prenatal glucocorticoids and long-term programming. Eur J Endocrinol. (2004) 151 (Suppl 3):U49–62. 10.1530/eje.0.151u049 15554887

[37] Lurbe E Ingelfinger J. Developmental and early life origins of cardiometabolic risk factors: novel findings and implications. Hypertension. (2021) 77:308–18. 10.1161/HYPERTENSIONAHA.120.14592 33390043

[38] Tyrrell JS Yaghootkar H Freathy RM Hattersley AT Frayling TM. Parental diabetes and birthweight in 236 030 individuals in the UK biobank study. Int J Epidemiol. (2013) 42:1714–23. 10.1093/ije/dyt220 24336895 PMC3887570

[39] Troy LM Michels KB Hunter DJ Spiegelman D Manson JE Colditz GA. Self-reported birthweight and history of having been breastfed among younger women: an assessment of validity. Int J Epidemiol. (1996) 25:122–7. 10.1093/ije/25.1.122 8666479

[40] Walton KA Murray LJ Gallagher AM Cran GW Savage MJ Boreham C. Parental recall of birthweight: a good proxy for recorded birthweight? Eur J Epidemiol. (2000) 16:793–6. 10.1023/A:1007625030509 11297220

