# Supplementary material for: Birth Weight and Adult Obesity Index in Relation to the Risk of Hypertension: A Prospective Cohort Study in the UK Biobank
Source: Front Cardiovasc Med. 2021 Jun 17;8:637437. doi: 10.3389/fcvm.2021.637437 (PMC8245673; doi:10.3389/fcvm.2021.637437)
Supplement: Supplementary file 1 [file Data_Sheet_1.pdf]

Supplementary Material

Supplementary Table 1. Basic characteristics of participants included and excluded in this study.

**Characteristic**	**Included**	**Excluded**
Participants, n (%)	199,893 (39.77)	302,727 (60.23)
Age, mean (SD), year	54.02 (8.05)	58.19 (7.69)
Female, No. (%)	126,619 (63.34)	146,765 (48.5)
White, No. (%)	193,594 (97.08)	279,104 (92.93)
Birthplace (England), No. (%)	160,703 (80.43)	229,797 (76.36)
Townsend deprivation index <0, No. (%)	148,425 (74.25)	209,224 (69.11)
BMI, mean (SD), kg/m2	26.53 (4.43)	28.03 (4.94)
Physical activity, mean (SD), MET-hours/week	44.81 (44.71)	43.72 (45.57)
Current smoking, No. (%)	20,547 (10.31)	32,432 (10.80)
Alcohol intake daily or almost daily, No. (%)	38,542 (19.29)	63,227 (20.99)
Family history of hypertension, No. (%)	96,299 (51.17)	142,997 (54.85)
Vegetable intake, mean (SD), serving/day	2.24 (2.13)	2.16 (2.17)
Fruit intake, mean (SD), serving/day	2.24 (1.54)	2.23 (1.66)

Abbreviation: BMI, body mass index (calculated as weight in kilograms divided by height in meters squared); MET, metabolic equivalent of task; SD, standard deviation.

Supplementary Table 2. Associations between birth weight and hypertension after excluding 5431 multiple birth subjects.

Hypertension	Birth weight quintiles, HR (95% CI)					*P* for non-linearity
	Q1 (<2.88kg)	Q2 (2.88-3.18kg)	Q3 (3.19-3.42kg)	Q4 (3.43-3.80kg)	Q5 (>3.80kg)	
No. of cases/ person years	2514/299738.01	2689/367218.68	2089/314365.67	2232/345281.93	2428/338204.99	NA
Model 1[a]	1.28 (1.21-1.35)	1.09 (1.03-1.15)	1.03 (0.97-1.09)	Ref (1)	1.00 (0.94-1.06)	<0.0001
Model 2[b]	1.25 (1.18-1.32)	1.12 (1.05-1.18)	1.04 (0.98-1.1)	Ref (1)	0.95 (0.9-1.01)	0.0002

Abbreviation: HR, hazard ratio; CI, confidence interval; Ref, reference; NA, not applicable.

[a] Model 1: adjusted for age, sex, maternal smoking, breastfeeding, birth place.

[b] Model 2: model 1 plus Townsend deprivation index, college or university degree, body mass index, physical activity (MET-hours/week), smoke status, alcohol intake frequency, vegetables and fruits intake, family history of hypertension, prevalent diabetes.

Supplementary Figure 1. Flowchart of study population.

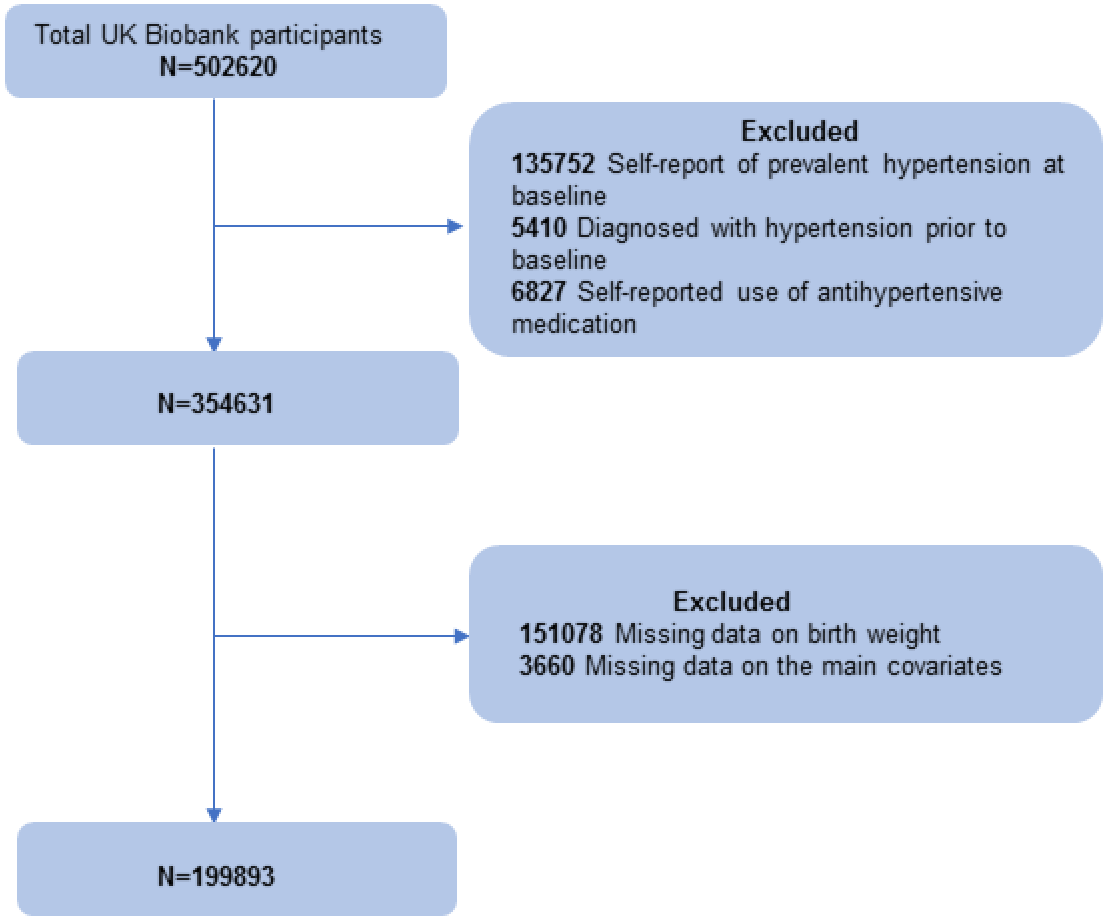

Supplementary Figure 2. **Stratified analysis of associations between birth weight and risk of hypertension according to sex (A), physical activity (B), smoking (C), breastfed (D) and maternal smoking (E).**

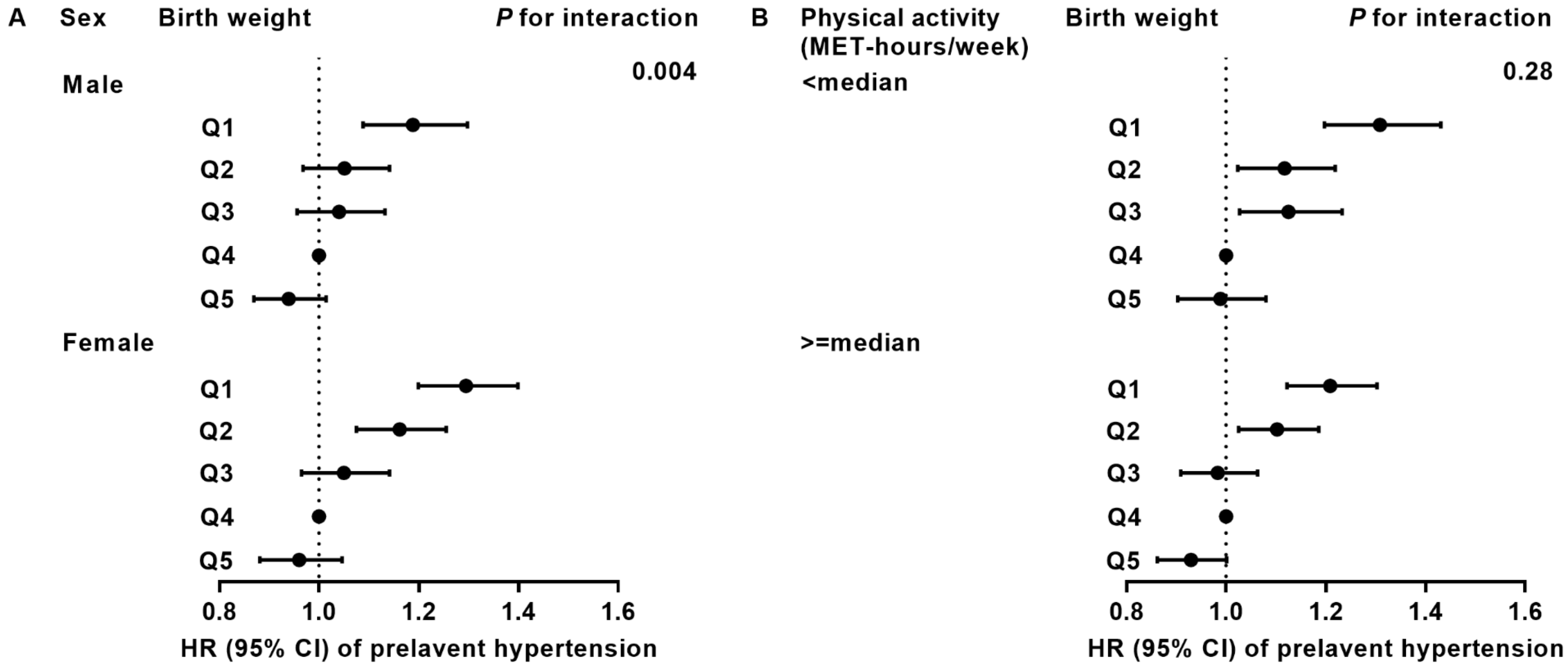

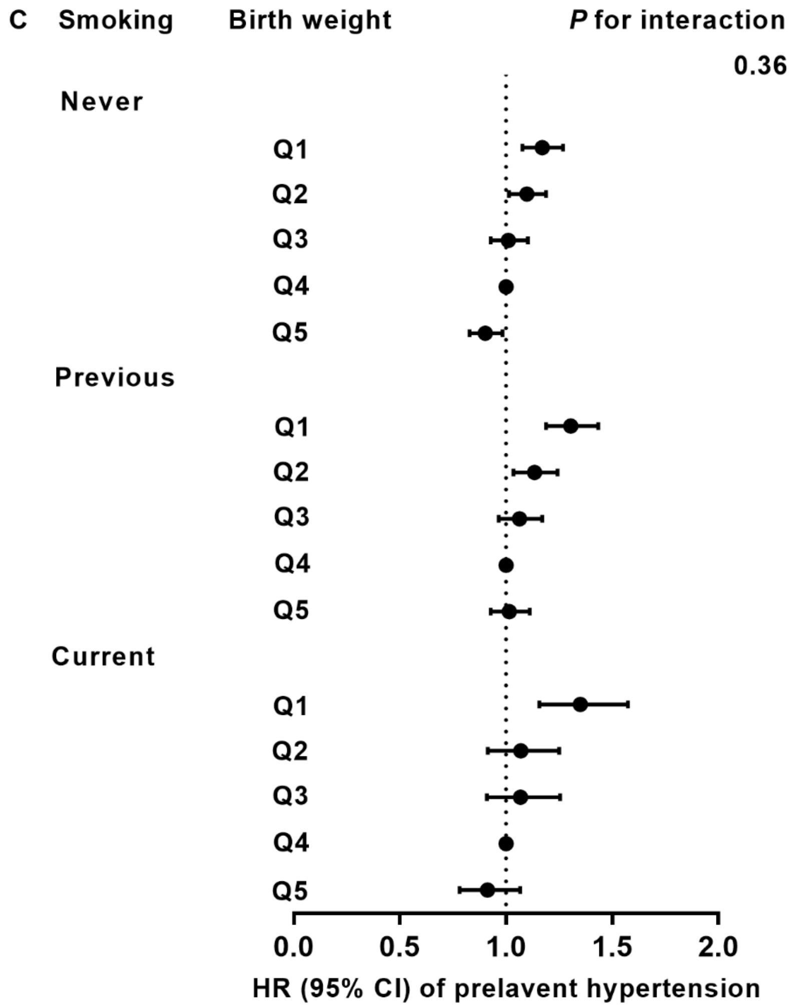

C Smoking
Birth weight
P for interaction
0.36
Never
Q1
Q2
Q3
Q4
Q5
Previous
Q1
Q2
Q3
Q4
Q5
Current
Q1
Q2
Q3
Q4
Q5
0.0
0.5
1.0
1.5
2.0
HR (95% CI) of prelavent hypertension

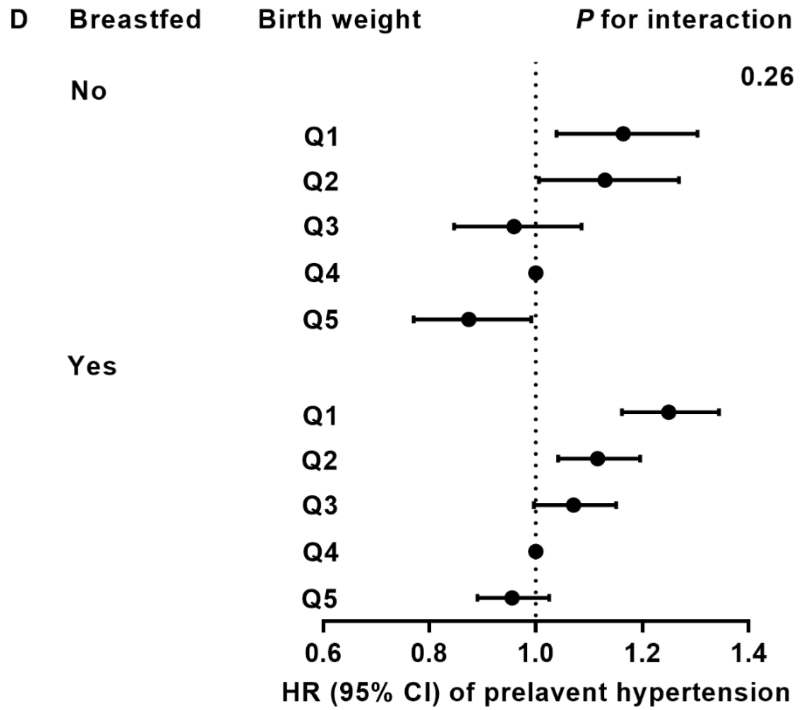

D Breastfed
Birth weight
P for interaction
0.26
No
Q1
Q2
Q3
Q4
Q5
Yes
Q1
Q2
Q3
Q4
Q5
0.6
0.8
1.0
1.2
1.4
HR (95% CI) of prelavent hypertension

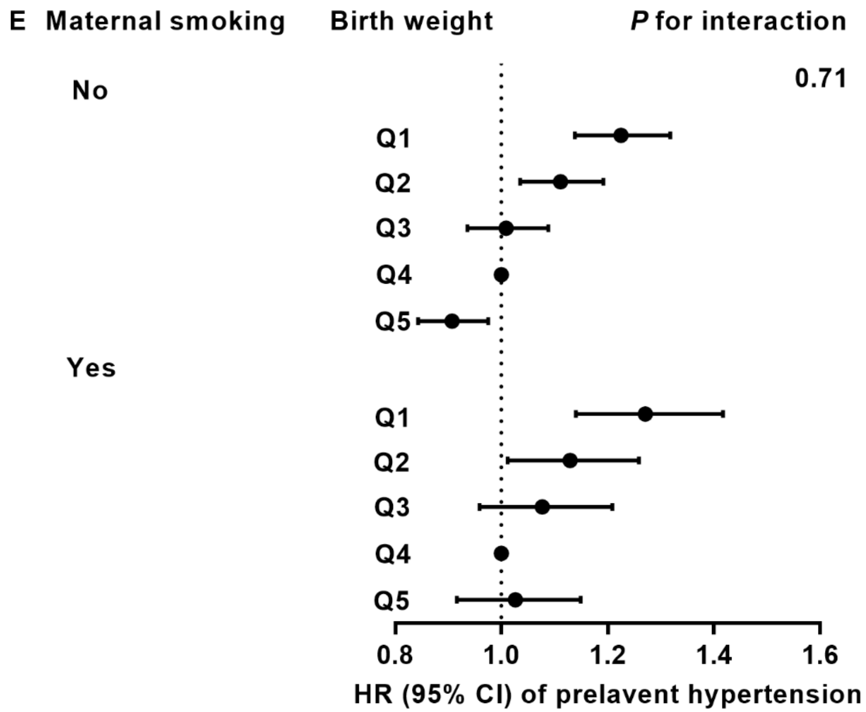

Note: Values are hazard ratios (95% confidence intervals) unless stated otherwise.

Supplementary Figure 3. Joint analysis of birth weight and waist-to-hip ratio (WHR) in relation to hypertension.

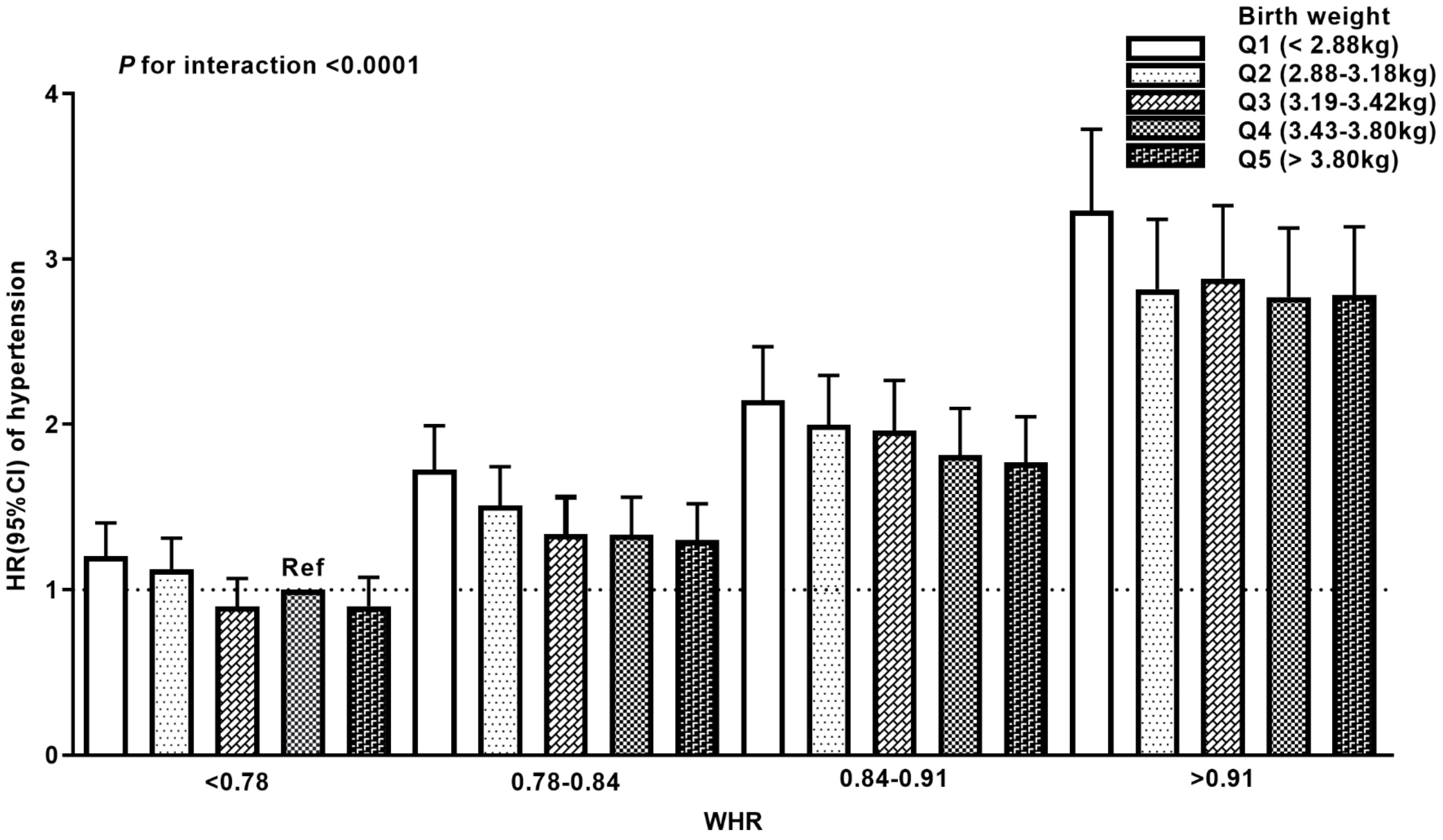

Note: Joint analysis of birth weight and WHR in the full model. The participants with birth weight in group Q5 (>3.80 kg) and WHR in adult lower than 0.78 were treated as the reference group (Ref). Error bars represent 95% confidence intervals of the hazard ratios.
